# The State of Child Health and Human Rights in Nepal

**DOI:** 10.1371/journal.pmed.0030203

**Published:** 2006-07-04

**Authors:** Sonal Singh, Erik Bøhler, Khagendra Dahal, Edward Mills

## Abstract

Time is running out for the children of Nepal, say the authors. These children will face an uncertain future unless their health and human rights are urgently addressed.

Nepal is one of the poorest countries in the world, with a per capita gross national product of US$240. Nearly 40% of the 25 million people living in Nepal do so on less than a dollar a day [ 1]. About 90% of the population lives in rural areas. The high-intensity (more than 1,000 deaths per year) conflict between the Communist Party of Nepal (Maoist) rebels and the government forces led by the Royal Nepalese Army has affected the health, education, and other rights of the most vulnerable members of society, especially women and children [ 2]. The conflict, which began in 1996, has resulted in widespread human rights violations by both parties as it draws the population into the conflict as both soldiers and victims.

In this article we examine the evidence on the current state of child health and human rights in Nepal ( Box 1). We argue that time is running out for the children of Nepal, as they face an uncertain future if their health and human rights concerns are not addressed by local governments, nongovernmental organizations (NGOs), and the international community in a timely manner. We also suggest possible solutions to the current problem.

## Bleak Indicators

In 1996, before the insurgency began, Nepal ranked 124 out of the 137 countries on the United Nations Development Programme's Human Development Index (HDI) [ 3, 4]. This index, which has a score of 0 to 1, gives a measure of longevity, health, education level, and standard of living. Nepal's HDI was 0.471 in 1996 [ 3, 4]. Although Nepal has since then moved from the rank of “low” to “medium” development countries (its HDI score in 2005 was 0.526 [ 5]), this apparent improvement has not improved the lives of most of the rural population. The armed conflict has eroded the tenuous gains in key development indicators [ 4].

The Nepal Demographic and Health Survey of 2001 found an infant mortality rate of 64 per 1,000 live births and a neonatal mortality rate of 39 per 1,000 live births [ 6]. There are widespread disparities in health services, life expectancies, education, and income between urban areas like the capital Kathmandu and the district headquarters on one side and the majority rural areas on the other. The under-5 mortality rate in urban areas is 93.6 per 1,000, whereas in rural and mountainous regions it increases to 147 per 1,000 and 201 per 1,000, respectively [ 6]. The maternal mortality rate is one of the highest in the world at 539 per 100,000 live births [ 5]. In rural Nepal more than 90% of birth deliveries are at home. Women face a one in 24 risk of dying during pregnancy and childbirth, and current levels of insecurity are increasing this risk further, as the conflict is hindering pregnant women from reaching hospitals for delivery [ 6, 7].

## Status of Child Health

One out of every 11 children in Nepal dies before reaching the age of five [ 6], and almost 70,000 children die yearly from preventable causes. One in two children is stunted or underweight, with little improvement in this situation since the 1990s [ 6]. A recent study estimated around 30,000 annual deaths due to diarrheal diseases in the country [ 8]. In a recent analysis, nearly 66% of schoolchildren in the northeastern part of Kathmandu Valley were found to have parasitic infections and nearly half of them had multiple parasitic infections [ 9]. Only one in four children in Nepal sees a health provider for illnesses [ 6]. Children are particularly vulnerable, because they are less likely to be taken long distances to health centers.

## Impact of the Conflict on the Health Sector

The Royal Nepalese Army controls the capital, Kathmandu, and the 75 district centers in the country, while many areas surrounding the district centers and rural areas are under Maoist control [ 10]. Families may be subjected to harassment if they attempt to leave or enter the Maoists' heartlands. A transport shutdown by the Maoist rebels in March 2005 held up the supply of vaccines, vitamin A, and de-worming drugs to nearly 3.6 million Nepalese children. Annually, some 12,000 children in Nepal would succumb to diseases without these essential medicines [ 10].

The delivery of health services has been disrupted in the far western regions and severely restricted in other parts of the country [ 11, 12]. Several community health posts have been destroyed and dozens of health-care workers have lost their lives. According to INSEC (Informal Sector Service Center), an independent human rights organization, 40 health posts were destroyed between January 2002 and December 2004 [ 10]. The Maoists destroyed the electrical supply of Okhaldhunga Hospital, a small hospital in remote eastern Nepal, in an attempt to harm a nearby army camp that got its electrical supply from the same power plant. As a result, operations such as caesarean sections were being performed under torchlight ( Figure 1). Health education programs conducted by the district public health offices and other private organizations are on the decline due to Maoist and government threats. Health-care workers fear a rise in communicable diseases and several organizations, including Médecins Sans Frontières, have had to scale back their activities in rural Nepal as a result of the insurgency.

**Figure 1:**
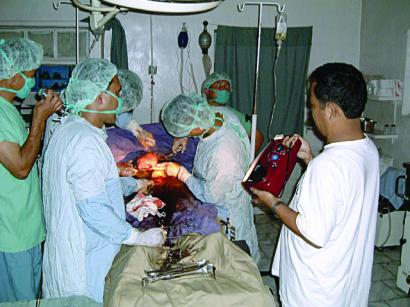
Operations at Okhaldhunga Hospital Being Carried Out without Electricity under Torchlight (Photo: Erik Bøhler)

The conflict has had a variable impact on coverage of primary-care posts in rural areas. [ 10]. In some Maoist-controlled areas, health post staff are threatened with reprisals if they do not stay at their posts. In other areas, staff have fled their posts since the beginning of the conflict, both for fear of their lives and because of heavy “taxation” of government employees by the Maoists. The government's directive that health professionals who provide treatment for injuries without appropriate notification can be prosecuted as supporters of terrorism has created a difficult scenario for health workers, who risk incarceration [ 13]. In April 2006, several physicians in Kathmandu were detained for taking part in peaceful demonstrations and two foreign physicians were deported for treating victims of the violence [ 14].

Although there is no study establishing a conclusive link between the conflict and mental disorders, a recent cross-sectional survey of 290 internally displaced people in Nepal found high rates of post-traumatic stress disorder (53.4%), anxiety (80.7%), and depression (80.3%) [ 15]. Psychiatrists and mental health hospitals have seen an increase in the number of patients in recent years (Okhaldhunga Community Hospital Public Health Unit, unpublished data). Data from Okhaldhunga Hospital in 2004 showed a general downward trend in attendance, probably due to transportation problems caused by the conflict, but an increase in consultations for mental disorders (Okhaldhunga Community Hospital Public Health Unit, unpublished data). Within this situation of uncertainty and conflict, hundreds of children raise themselves, due to the loss of their parents and relatives [ 16]. Among students, 74% percent in Maoist-affected areas fear that the rebels or government forces might abduct them [ 17]. There is a general lack of hope, especially among the youth. Some of them have left the countryside. Others have joined the insurgents or developed reliance on drugs and alcohol [ 15].

## Children Lack Food and Education

Children face food insecurities due to frequent blockades and cutbacks in local food production caused by the exodus of merchants from rural areas, lack of access to markets, and the displacement of able members of some households [ 18]. This food insecurity is not evident in Kathmandu, but is clear in the rural areas. The very high prevalence of babies who are “low weight for gestational age” means that newborn children are already at risk [ 19]. The malnutrition situation is particularly serious in many parts of the midwestern region, which are badly affected by the conflict, with Humla district having the highest rate of malnourishment in the country [ 20]. A recent survey by the NGO Terre des Hommes among internally displaced children under three years of age in four Village Development Committees in a western Terai district found that 59% of children were underweight and 15.9% had wasting [ 21].

The conflict has also deprived Nepalese children of education. Prior to the conflict, access to education in Nepal was extremely limited for girls, members of the lower castes, and other disadvantaged groups [ 22]. Currently one out of every five children aged six to ten does not attend school [ 23]. Nearly 700 private schools have closed down since 1996. Even in districts where schools are open, the continuing series of strikes and blockades has reduced the time children can go to school [ 22]. Schools in rural areas are under-attended by students and teachers due to fear, insecurity, and displacement [ 18]. Schools have been bombed and attacked [ 18]. Mines have been placed in and around schools and playgrounds [ 22]. Schools have been used as grounds for child recruitment and abduction of teachers, or turned into barracks and used for political meetings [ 18].

## HIV/AIDS in Children

Although the incidence of HIV in children in Nepal is low, WHO/UNAIDS (World Health Organization/United Nations Programme on HIV/AIDS) estimates that there were 940 children living with HIV and nearly 13,000 children orphaned due to AIDS at the end of 2003 [ 24]. The reality of the situation is unclear, as many have been trafficked to India for sex work [ 24, 25]. A study by General Welfare Pratishthan, an NGO working on the prevention of HIV and STDs, postulated that the current conflict may have pushed a large number of young girls from rural villages to urban areas in search of food, shelter, and security who later end up involved in prostitution for subsistence [ 26]. This may fuel the spread of HIV among young girls.

## Children and Human Rights

Since the end of the cease-fire in 2003, there has been a steady increase in human rights violations against Nepalese children by both parties to the conflict. The National Human Rights Commission estimates that more than 500 children have been killed in the conflict [ 27]. Although it is extremely difficult to assess the complete extent of child soldiering in Nepal, the use of child soldiers is common by both parties to the conflict [ 28, 29]. Girls are raped and subjected to other forms of sexual violence by both Maoists and government forces; survivors of gender-based violence often remain silent due to a lack of protection [ 18].

Nepal holds the dubious distinction for the highest number of “disappearances” in 2004. Children under age 18 have “disappeared” and have been arbitrarily detained by government forces [ 18]. According to INSEC, nearly 3,000 people were killed and about 26,000 people were abducted in Nepal in 2004 [ 30]. While accurate estimates are hard to come by, Child Workers in Nepal, a local NGO, estimates that in 10 years of the insurgency 27,323 children have been abducted, while the state security forces have arrested 229 children [ 31]. From January to August 2005 the insurgents are said to have abducted 11,802 children while the security forces have arrested 17 children [ 31]. There have been hardly any prosecutions of government officials or Maoists for their involvement in disappearances [ 32].

## Recent Progress

In recent years there has been some progress in finding indigenous solutions to improving the health of children despite the difficult circumstances. A survey of primary health-care services in central Nepal found that health care for those with resources had improved considerably, but massive privatization had severely limited access for the poor [ 33]. Improvements in maternal literacy have also been shown to improve health behavior in Nepal [ 34]. The vitamin A program was successfully implemented in the remotest districts of Nepal with community participation. A recent cluster randomized trial assessed the use of simple interventions, such as participation in women's groups at the community level and monthly women's group meetings with female facilitators to identify local perinatal problems and strategies to address them. The trial showed a reduction in neonatal mortality rates from 36.9 per 1,000 to 26.2 per 1,000 [ 35]. Women in the intervention clusters were more likely to have antenatal care, institutional delivery, trained birth attendance, and hygienic care than were controls.

The recently released Millennium Development Goals Report is optimistic that Nepal will meet the goals of: halving the proportion of people living below the national poverty line; reducing the under-5 mortality rate by two-thirds; achieving gender equality; improving maternal health; and ensuring environmental sustainability by 2015 [ 36]. But in the current political and developmental climate, it seems unlikely that the child health goals will be met. The goals of achieving universal primary education and reversing the spread of HIV/AIDS are also unlikely to be realized [ 36].

## The Road Ahead

In a country as diverse as Nepal, no single solution can address the current problems facing child health. Sparsely populated mountains with Tibetan language and nomadic traditions pose different challenges than the densely populated Terai regions, which have similarities to India. Unless the government starts introducing policy changes aimed at making valuable health services accessible for the poor, children's health in this country will not improve. Systematic capacity building to support the Female Community Health Volunteers or “Sevikas” is the best hope for sustaining the momentum of programs that promote health equity through interventions such as family planning, polio prevention, and vitamin A supplementation [ 33]. Improving the quality of services provided at *existing* health centers, rather than increasing the number of centers, is an effective way to improve child health in Nepal [ 37]. In the regions most affected by the conflict, the health situation should be monitored by using sentinel site surveys (surveys of representative sites to collect health related information using UN teams of experts) and participatory appraisal (a process which invites people to explore the issues that affect them to find realistic and feasible solutions to their problems). Such monitoring requires commitments by both Maoist and government authorities to allow survey teams to assess the current health and human rights situation and respect demographic and health surveys.

In order to sustain child development, it is imperative that both parties halt all violations against children in Nepal and uphold international humanitarian law and human rights [ 38]. Both parties have to refrain from drawing children into the violence and should view children as “the zone of peace” and schools and health posts as “weapon-free zones.” The agreement between the Nepalese government and the UN Human Rights Commission to set up an international monitoring mechanism to protect human rights is a step forward [ 39]. Both parties should invite international observers such as the United Nations to instigate, conduct, and monitor a peace process [ 40]. There is also an urgent need to implement a human rights accord which abides by the Geneva Conventions and commits both the government and Maoists to respect clear human rights standards and accept human rights monitoring [ 40].

The government of Nepal should conduct independent, impartial, public investigations into atrocities against children and allocate the necessary funds to ensure that young people have access to adequate health care and education [ 18]. Immediate emergency support should be provided to children affected by the conflict by the state, government organizations, and NGOs, and efforts should be made to rehabilitate child survivors [ 38]. Nongovernmental organizations and civil society must strive to enforce existing legislation protecting children and also address the issues of child soldiers, child labor, and trafficking in children affected by the conflict [ 41].

The king of Nepal reinstated the parliament in April 2006 after nationwide protests [ 42]. The Maoist rebels have signed a code of conduct with the representatives of the government, raising hopes for a peaceful solution to the conflict in Nepal.

The international community, which provides 60% of Nepal's development budget, has a larger role to play in protecting the rights of children. The work of the international community has become more critical as the freedom of the media has been curtailed by the promulgation of an ordinance that ensures the state's direct control over the private and free media. Another law to restrict the independent work of the NGOs may handicap the actions of organizations involved in health and human rights issues [ 43]. The international community needs to raise its voice against the atrocities levied against children by both conflicting parties. It needs to provide enough resources to meet the basic needs of the children and support developmental programs, but ensure that foreign support does not fund military exercises or increase child health violations, in order to prevent the children of Nepal from becoming another “lost generation.”

**Box 1. Search Strategy**

In order to identify information for our article, we searched Medline, Google Scholar, POPLINE, World Health Organization reports, United Nations Development Programme–Nepal reports, the UN Office for the Coordination of Humanitarian Affairs (IRINnews) Web site, and Eldis Development Gateway, using the terms “Nepal,” “child health,” “human rights,” and “conflict.” We searched reports of several non-governmental organizations including Save the Children, Family Health International, and CARE Nepal. We selected articles for inclusion based on their relevance to the topic and their ability to advance our understanding of the impact of the conflict on child health and human rights. In addition, we identified unpublished research through contacts with authors and experts in the field. We acknowledge the lack of epidemiological studies directly linking the conflict to child health care in Nepal; documentation on crucial aspects of the conflict was often impossible to obtain and we rely on newspaper articles and eyewitness accounts at times. We drew from the collective expertise of the authors (SS, EB, KD) involved in delivering health care in Nepal and incorporated suggestions from international health experts and NGOs active in this field.

## Abbreviations

HDI: Human Development Index
NGO: non-governmental organization
